# MGH Whole Slide Imaging Teleconsultation Practice in Dermatopathology

**DOI:** 10.1155/2014/347147

**Published:** 2014-11-19

**Authors:** Nicholas C. Jones, Rosalynn M. Nazarian, Lyn M. Duncan, David C. Wilbur

**Affiliations:** Department of Pathology, Massachusetts General Hospital and Harvard Medical School, Boston, MA 02114, USA

## Background

Nonsubspecialized pathologists frequently request expert consultation in challenging dermatopathology cases. Traditional consultation practice utilizing shipment of glass slides is costly, slow, and of limited educational benefit to the referring physician. Whole slide imaging (WSI) has been suggested as a potential method of overcoming these limitations in the current glass slide consultation practice, but there have been concerns regarding the adequacy of image quality for interpretation of challenging dermatopathology cases. We aimed to investigate the performance of WSI in challenging dermatopathology consult cases.

## Method

52 consecutive clinical consultation dermatopathology cases sent from a community hospital to an academic medical center were sampled and diagnosed by traditional microscopic examination and by whole slide image examination. Matched pairs of diagnoses were evaluated for diagnostic accuracy rates via a masked adjudication process.

## Results

Two of 52 cases (3.8%) had major discrepancies. After adjudication the WSI diagnosis was preferred in one case and the glass slide diagnosis was preferred in the other. 13 of 52 cases (25%) had minor discrepancies, with the WSI diagnosis preferred in 6 cases and the glass slide diagnosis preferred in 4 cases and with no preference in 3 cases. Differences in diagnosis were primarily due to interobserver variability and thresholding inherent in challenging dermatopathology consult cases and not due to image quality.

## Conclusions

Overall, the sampled accuracy rates of both WSI and glass slide techniques were equivalent. These results suggest that WSI may be feasible for even challenging dermatopathology consultation cases.

